# Transcriptome and physiological analyses provide insights into the leaf epicuticular wax accumulation mechanism in yellowhorn

**DOI:** 10.1038/s41438-021-00564-5

**Published:** 2021-06-01

**Authors:** Yang Zhao, Xiaojuan Liu, Mengke Wang, Quanxin Bi, Yifan Cui, Libing Wang

**Affiliations:** 1 State Key Laboratory of Tree Genetics and Breeding, Research Institute of Forestry, Chinese Academy of Forestry, 100091 Beijing, China; 2 State Key Laboratory of Plant Genomics, Institute of Genetics and Developmental Biology, Chinese Academy of Sciences, 100101 Beijing, China; 3 University of Chinese Academy of Sciences, 100039 Beijing, China

**Keywords:** Drought, Metabolomics

## Abstract

Plantations and production of yellowhorn, one of the most important woody oil and urban greening trees widely cultivated in northern China, have gradually become limited by drought stress. The epicuticular wax layer plays a key role in the protection of yellowhorn trees from drought and other stresses. However, there is no research on the mechanism of wax loading in yellowhorn trees. In this study, we investigated the anatomical and physiological characteristics of leaves from different germplasm resources and different parts of the same tree and compared their cuticle properties. In addition, the different expression patterns of genes involved in wax accumulation were analyzed, and a coexpression network was built based on transcriptome sequencing data. Morphological and physiological comparisons found that the sun leaves from the outer part of the crown had thicker epicuticular wax, which altered the permeability and improved the drought resistance of leaves, than did shade leaves. Based on transcriptome data, a total of 3008 and 1324 differentially expressed genes (DEGs) were identified between the sun leaves and shade leaves in glossy- and non-glossy-type germplasm resources, respectively. We identified 138 DEGs involved in wax biosynthesis and transport, including structural genes (such as *LACS8*, *ECH1*, and *ns-LTP*) and transcription factors (such as MYB, WRKY, and bHLH transcription factor family proteins). The coexpression network showed a strong correlation between these DEGs. The differences in gene expression patterns between G- and NG-type germplasm resources under different light conditions were very clear. These results not only provide a theoretical basis for screening and developing drought-resistant yellowhorn germplasm resources but also provide a data platform to reveal the wax accumulation process of yellowhorn leaves.

## Introduction

Yellowhorn (*Xanthoceras sorbifolium* Bunge) is one of the most important species of woody oil and greening trees and has been widely cultivated in northern China in recent years. As an endemic species to China, yellowhorn has not only high medicinal and ornamental value but also important socioeconomic value due to its high fatty acid content^1^. Notably, the seed oil of yellowhorn contains nervonic acid, which has an influence on memory improvement and is almost nonexistent in other plants^2^. In the face of a global scarcity of water resources, drought has already become a primary factor limiting the productivity and geographical distribution of yellowhorn. Thus, discovering the stress resistance features of yellowhorn and their underlying mechanisms has become an increasingly important issue.

Fortunately, plants, including yellowhorn, have evolved various strategies to respond and adapt to drought stress through their long evolutionary history. Among these strategies, cuticles form the primary essential barrier for decreasing nonstomatal water loss under drought stress^3^. The cuticle is a unique structure developed by land plants. Structurally, the cuticle is made of the outermost epicuticular wax layer; the middle cutin layer, which is embedded with intracuticular waxes; and the inner cutin layer^3^. Cuticular wax, as a major component of plant cuticles, is a lipophilic layer and is mainly composed of very long-chain fatty acids (VLCFAs, C20–C34) and their derivatives, aldehydes, primary and secondary alcohols, ketones, alkanes, unsaturated fatty alcohols, and wax esters^4^. Increased drought tolerance by the enhancement of cuticular wax accumulation has been confirmed in many studies^5–7^.

The process of cuticular wax accumulation is multistep and precisely regulated by the external environment. The synthesis of VLCFAs requires catalyzing enzymes such as acyl-CoAs, fatty acid reductase (FAR), and fatty acid elongase (FAE) complexes, which include β-ketoacyl-CoA synthase (KCS), β-ketoacyl-CoA reductase (KCR), β-hydroxyacyl-CoA dehydratase (HCD) and enoyl-CoA reductase (ECR), and lipid transfer proteins such as ATP-binding cassette (ABC) transporters^8^. Cuticular wax biosynthesis is known to be a complex multilevel process and includes transcriptional, posttranscriptional, and posttranslational regulation^8,9^. Several transcription factors, such as DECREASE WAX BIOSYNTHESIS (DEWAX), WAX INDUCER 1 (WIN1)/SHINE 1 (SHN1), and MYB16, have revealed that the transcriptional regulatory mechanism is a major contributor to wax biosynthesis^10–13^.

However, previous studies on the response of yellowhorn to drought were mostly limited to the physiological level, and there have been few investigations of leaf cuticles^14^, especially at the molecular level. The molecular mechanisms of yellowhorn leaf cuticular wax accumulation remain largely unknown. In this study, based on two yellowhorn germplasm resources with contrasting cuticular wax thicknesses, the differences in cuticular wax accumulation were investigated through a combination of physiological, microstructural, and comparative transcriptomics analyses. This research aims to elucidate the wax load differences between different types of yellowhorn germplasm resources at the morphological, physiological, and molecular levels. This study may also provide new insights into the selection of drought-resistant yellowhorn resources and help us better understand the underlying biological process of leaf cuticular wax load.

## Results and discussion

### Sun leaves have higher leaf wax accumulation than shade leaves

**Fig. 1:**
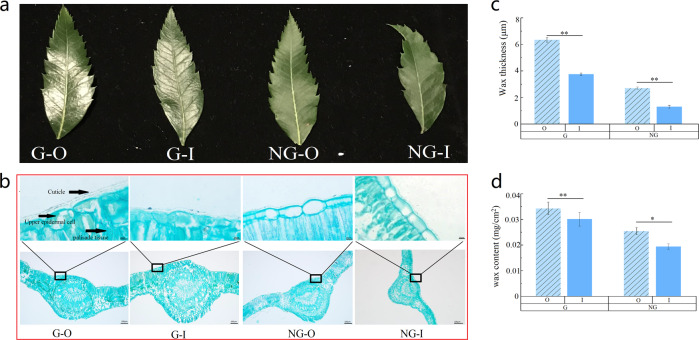
Comparison of cuticular wax accumulation. **a** Photographic images of G–O-, G–I-, NG–O-, and NG–I-type leaves. **b** Anatomical structure characterization of G–O-, G–I-, NG–O-, and NG–I-type leaves. **c** Analysis of epidermal cuticle thickness. (**d**) Comparison of wax contents in the leaf epidermis. Error bars represent standard error. ‘*’ on error bars indicates significant differences at *p* < 0.05 among two groups, ‘**’ indicates significant differences at *p* < 0.01 among two groups

Based on the differences in morphology, we selected glossy (G)-type yellowhorn germplasm resources, which possess a glossy, dark-green leaf surface in light, and non-glossy (NG)-type yellowhorn germplasm resources, which exhibit a non-glossy, gray-green leaf surface, as plant materials (Fig. 1a). Three plants were selected from G- and NG-type yellowhorn germplasm resources. Sun leaves (O) were selected from the outer part of the crown and exposed to direct rays of the sun. Control shade leaves (I) were collected from the lower or inner part of the crown and were subjected to dense shade. The light intensity in the lower to middle crown was almost 1/10 of that at the outer part of the crown (Supplementary Fig. S1). Similar differences were also observed in leaves from different positions on the same plant (Fig. 1a). To further compare the wax accumulation in the different types of leaves, anatomical characterization of the leaves was performed. Under both sun and shade conditions, the cuticle thickness of the adaxial (facing the stem) leaf surface in G-type germplasm resources was extremely significantly (*p* < 0.01) higher than that of NG-type germplasm resources (approximately two times). The sun leaves (O), which were collected from the outer part of the crown and received full sunlight, showed a significantly (*p* < 0.01) thicker cuticle than shade leaves (I) in both germplasm resources, indicating that greater light intensity and induced radiation results in more wax accumulation on leaves (Fig. 1b, c). To quantify changes in the wax content, wax from leaves was extracted with chloroform, and the content was calculated by dividing the extracted wax weight by the leaf area (LA). The results from different samples are presented in Fig. 1d. In both germplasm resources, the wax contents of sun leaves were higher (~13.3% in the G type and 24.2% in the NG type) than those of shade leaves. In addition, the coverage of superficial wax on the adaxial leaf surface was visualized by SEM (Supplementary Fig. S2). The wax of the G-type leaves had a uniform and dense appearance, while it was relatively irregular and sparse in the NG-type. In particular, only a small amount of wax of the NG–I leaf was found on the film, while for others, it could easily be detected. These results indicate that enhancing the strength of light increases the leaf surface wax load.

### Drought tolerance improved by altered cuticle properties

**Fig. 2:**
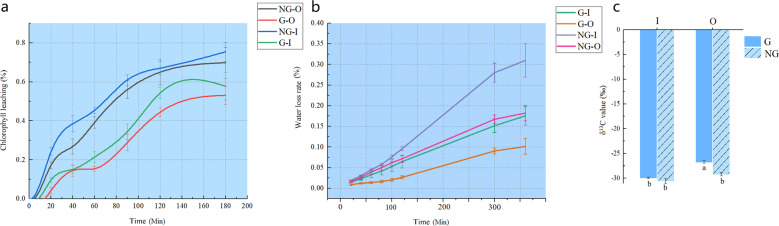
Altered cuticular permeability and drought resistance of the four types of leaves. **a** Chlorophyll leaching assays with mature leaves of G–O, G–I, NG–O, and NG–I immersed in 80% ethanol for different time intervals. **b** Water-loss rate of detached leaves of the four types. The *x*-axis is the scale of different time points, and the *y*-axis is the percentage of free water loss from the leaves. Each point is the mean ± standard error. **c** Comparison of δ^13^C values of different samples. Different lowercase letters represent significant differences

The most important physiological function of the cuticle is to reduce nonstomatal water loss of leaves under drought conditions. To investigate whether the cuticular properties of the leaves were different, chlorophyll leaching assays were conducted by submerging leaves in 80% ethanol at different time points in the dark after stomatal closure treatment. Among the four samples, the two lines of G-type germplasm resources (G–O and G–I) showed lower chlorophyll leaching at six time points than NG-type germplasm resources (NG–O and NG–I). In addition, the shade leaves lost their chlorophyll faster than the sun leaves after 40 min of treatment. At the 180 min time point, the shade leaves (G–I and NG–I) leached 57.80% and 75.42% of their total chlorophyll, respectively, while the sun leaves (G–O and NG–O) leached only 53.06% and 69.77%. The chlorophyll leaching assays revealed a major difference in the cuticle permeability of sun leaves and shade leaves from G- and NG-type germplasm resources due to their different levels of wax accumulation (Fig. 2a).

Similarly, for the water-loss assay, the loss of epidermal water vapor was slower in sun leaves and faster in shade leaves in both types of germplasm resources (Fig. 2b). Water-loss rates of leaves from G-type germplasm resources were lower than those of NG-type germplasm resources at various time points. At the 360 min time point, the detached leaves of the shade samples lost 17.56% and 30.98% water, while the leaves from the sun leaves lost 10.15% and 18.23% (Fig. 2b), respectively. The speed of water loss in the different materials was as follows: NG–I (5.4%/h) > NG–O (3.6%/h) > G–I (3%/h) > G–O (1.8%/h). Simple linear correlation analysis indicates that the water loss speed is related to the wax thickness (*R*^2^ = 0.92) and wax content (*R*^2^ = 0.96) (Supplementary Fig. S3). These results suggest that cuticular wax has an important role in protecting leaves from water loss; furthermore, drought resistance and cuticular permeability of leaves could be altered with an increase in wax accumulation due to the variation in light conditions. In addition, water use efficiency (WUE) was measured to examine drought resistance by determining the δ^13^C value (Fig. 2c). The results of determining ^13^C showed that the WUE of sun leaves was significantly (*p* < 0.05) higher than that of shade leaves in G-type germplasm resources, while the WUE of sun leaves was slightly higher than that of shade leaves in NG-type germplasm resources. These results indicate that higher cuticular wax accumulation could improve the plant’s drought resistance by altering the leaf permeability.

### Identification of differentially expressed genes

To further elucidate the molecular basis for the differential wax accumulation in leaves with different wax contents, we conducted comparative transcriptome analysis based on Illumina sequencing technology. Twelve libraries were created and sequenced from three biological replicates for each group of sun leaves (O) and shade leaves (I) from the G- and NG-type germplasm resources. After quality control and filtering for each sample, more than 4.4 million clean reads were provided. The genome alignment rates of the 12 libraries ranged from 88.81 to 96.65%. Moreover, ~81.09% of the high-quality reads of each library could be uniquely mapped to yellowhorn (Table 1).

**Table 1:** Overview of sequencing quality and alignments

Samples	Raw reads number	Clean reads number	alignment rate (%)	Uniq mapped paired reads number	Uniq mapped rate (%)	% ≥ Q30
G–I-1	########	########	90.74	18587163	78.60	93.55
G–O-1	########	########	93.48	17698972	78.41	94.17
G–I-2	########	########	93.22	18742643	81.52	93.45
G–O-2	########	########	96.65	19198612	84.83	93.42
G–I-3	########	########	94.37	20530127	81.94	93.78
G–O-3	########	########	95.03	18126722	81.51	92.88
NG–I-1	########	########	88.81	17052026	76.71	93.61
NG–O-1	########	########	93.77	18771576	80.76	93.27
NG–I-2	########	########	93.51	19433496	81.22	93.68
NG–O-2	########	########	96.17	17847879	83.48	93.81
NG–I-3	########	########	91.50	17693229	80.12	93.83
NG–O-3	########	########	95.76	18317915	83.99	93.81

**Fig. 3:**
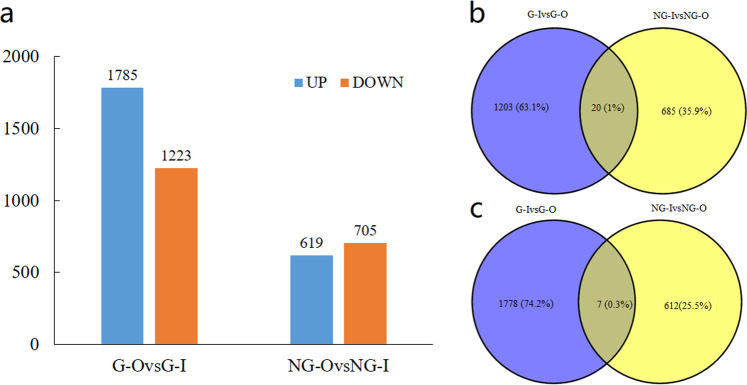
Summary of DEGs between the 4 leaf types. **a** Numbers of DEGs in pairwise comparisons of the four samples; the Venn diagram shows the number of **b** downregulated, and **c** upregulated DEGs in G–I vs. G–O and NG–I vs. NG–O

We used the protocol described by Pertea^15^ to analyze RNA-seq data using HISAT2, StringTie and Ballgown in R packages. After comparisons of the fragments per kilobase of transcript length per million mapped reads (FPKM) values for each gene between sun leaves and shade leaves (G–O vs. G–I and NG–O vs. NG–I), DEGs were identified. Finally, the results (*p* < 0.05) were defined as significant differences in gene expression. The number of DEGs was as follows: 1785 up- and 1223 downregulated DEGs in G–I vs. G–O and 619 up- and 705 downregulated DEGs in NG–I vs. NG–O (Fig. 3a). The Venn diagram shows the overlap of up- and downregulated DEGs (Fig. 3b, c). Among them, only 27 DEGs were common in the G and NG types, which indicates that there are different response mechanisms involved in the regulation of wax accumulation in G- and NG-type germplasm resources.

### Functional classification of the DEGs by enrichment analysis

To identify the major functional categories of the DEGs in the G and NG types, Gene Ontology (GO) enrichment analysis was carried out using OmicShare tools. Upregulated DEGs in the G (G–I vs. G–O) and NG (NG–I vs. NG–O) types were categorized into 51 and 47 GO terms, respectively (Supplementary Fig. S2). In the biological process category, the largest class was metabolic process, and 619 and 221 DEGs were enriched in the G (G–I vs. G–O) and NG (NG–I vs. NG–O) types, respectively. Among the molecular function categories, 570 and 191 DEGs in the G (G–I vs. G–O) and NG (NG–I vs. NG–O) types were enriched in catalytic activity. In the cellular component category, 493 and 183 DEGs in the G (G–I vs. G–O) and NG (NG–I vs. NG–O) types, respectively, were enriched in the cell category, which was the largest class. For downregulated DEGs in the G (G–I vs. G–O) and NG (NG–I vs. NG–O) types, DEGs were categorized into 47 and 44 GO terms, respectively (Supplementary Fig. S2). In the biological process, molecular function and cellular component categories, the largest enrichment classes were metabolic process, catalytic activity, and cell class, respectively, which was consistent with the results for the upregulated DEGs.

We performed Kyoto Encyclopedia of Genes and Genomes (KEGG) enrichment analysis to further understand the molecular interactions of DEGs in the G (G–I vs. G–O) and NG (NG–I vs. NG–O) types. For upregulated DEGs, the DEGs in the G (G–I vs. G–O) and NG (NG–I vs. NG–O) types were enriched in 18 pathways, and the main enriched DEGs were involved in “Global and overview maps”, “Folding, sorting and degradation” and “Carbohydrate metabolism” (Fig. S2c). Lipid metabolism was enriched in 20 and 9 DEGs in the G (G–I vs. G–O) and NG (NG–I vs. NG–O) types, respectively. However, there were major differences in the KEGG enrichment of downregulated DEGs. The DEGs were enriched in 41 and 17 pathways for the G (G–I vs. G–O) and NG (NG–I vs. NG–O) types (Fig. S2d), respectively. The two types had identical enrichment in “Global and overview maps”, “Signal transduction”, “Translation”, and “Folding, sorting and degradation”. Cell growth and death, cellular community, environmental adaptation, signal transduction, membrane transport, energy metabolism, metabolism of terpenoids and polyketides, and biosynthesis of other secondary metabolites were uniquely enriched in the G (G–I vs. G–O) type. Notably, lipid metabolism enriched 13 and 5 DEGs in the G (G–I vs. G–O) and NG (NG–I vs. NG–O) types, respectively.

### Differentially expressed genes involved in wax accumulation

To identify the key genes involved in cuticular wax load in yellowhorn, the gene expression patterns of DEGs related were analyzed based on their function. For the enrichment analysis, we selected 28 DEGs involved in UV and heat stress response, fatty acid metabolism and lipid transport and metabolism pathways, such as the fatty acid biosynthetic process, long-chain fatty acid metabolic process, very long-chain fatty acid metabolic process, lipid metabolic process, and ABC transporter family.

**Fig. 4:**
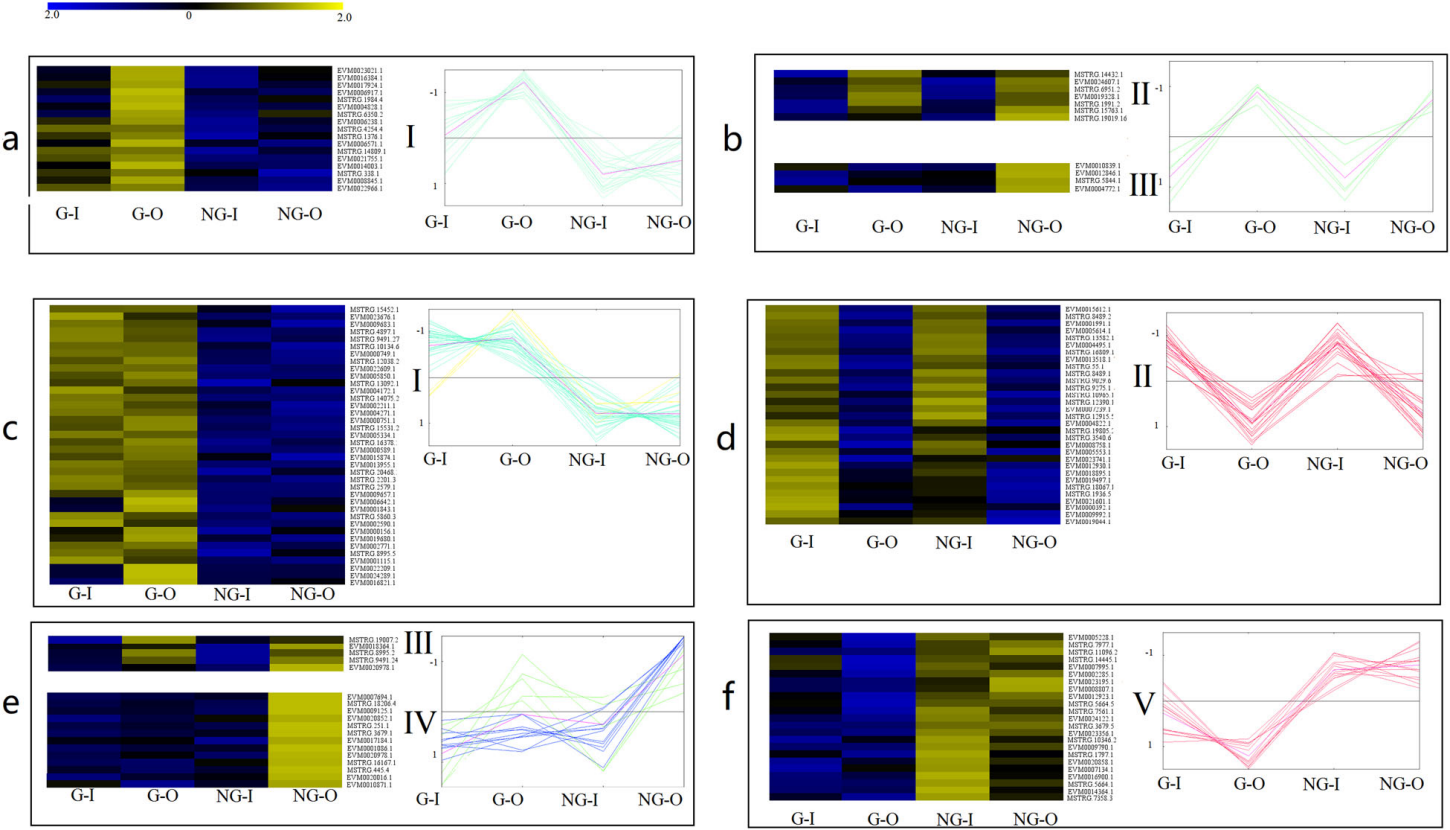
Heatmap diagrams showing the expression patterns of DEGs involved in lipid metabolism. **a** Heatmap diagrams of DEGs clustered into the first group (I). The cyan line represents the expression patterns of each DEG, and the pink line represents the overall trend of these DEGs. **b** Heatmap diagrams of DEGs clustered into the second and third groups (II). The light green line represents the expression patterns of each DEG, while the pink line represents the overall trend of these DEGs. **c** Heatmap diagrams of TFs clustered into the first group (I). The cyan and yellow lines represent the expression patterns of DEGs upregulated in the G type and G–O type, respectively, while the pink line represents the overall trend of these DEGs. **d** Heatmap diagrams of TFs clustered into the second group (II). **e** Heatmap diagrams of TFs clustered into the second (III) and third groups (IV). The light green and dark blue lines represent the expression patterns of TFs clustered in groups (III) and (IV), respectively, while the pink line represents the overall trend of these TFs. **f** Heatmap diagrams of DEGs clustered into group (V)

According to their expression trends, the 28 DEGs could be divided into three groups (I–III) (Fig. 4 and Supplementary Table S1). Seventeen genes whose expression was significantly upregulated in the G-type germplasm resources were fit into the first group (I) (*p* < 0.05), including, omega-3 fatty acid desaturase (*FAD*, EVM0006238.1) and fatty acid hydroxylase (*WAX2*, MSTRG.4254.4), which was identified as being involved in wax biosynthesis in a previous study^16^, ABC transporter A family member 7 (EVM0022966.1), ABC transporter I family member 11 (MSTRG.1376.1), and vacuolar protein sorting-associated protein 24 (EVM0021755.1). In particular, 10 genes were upregulated in the G–O type, such as long-chain acyl-CoA synthetase 7 (*LACS7*, EVM0017924.1)^17^, enoyl-CoA hydratase 2 (*ECH2*, EVM0006917.1), acetyl-CoA acetyltransferase (*MIF*, MSTRG.1984.4), putative lipid-transfer protein DIR1 (EVM0023021.1), vacuolar protein sorting-associated protein 22 (EVM0006571.1), heat shock 70 kDa protein (MSTRG.6350.2), and small heat shock protein (EVM0004828.1). The second group (II) consisted of seven genes that showed significantly upregulated expression in both the G–O and NG–O types (*p* < 0.05): enoyl-CoA hydratase (*ECH*, EVM0024607.1) and long-chain acyl-CoA synthetase 8 (*LACS8*, MSTRG.1991.2), which are involved in fatty acid metabolism and wax accumulation in *Arabidopsis*^12^; ultraviolet-B receptor UVR8 (MSTRG.6951.2), a UV-B photoreceptor that mediates ultraviolet protection^18^; and ABC transporter I (MSTRG.14432.1), ABC transporter G family member 36 (MSTRG.19019.16), and ABC transporter G family member 14 (EVM0019328.1), which were highly expressed in both the G–O and NG–O types. The third group (III) had 4 genes that were more highly expressed in the NG–O type (*p* < 0.05): acyl-[acyl-carrier-protein] desaturase (EVM0012846.1), which has also been identified in liquid wax ester biosynthesis of Jojoba^19^; vesicle-associated membrane protein 711 (EVM0010839.1); ABC transporter C family member 10 (MSTRG.5844.1); and universal stress protein A (EVM0004772.1).

Many studies have found that wax accumulation is governed by transcription factors (TFs), the master regulator proteins that control how genes are turned off and on. In our study, 110 differentially expressed TFs in G- and NG-type germplasm resources were identified and divided into five groups (Fig. 4 and Supplementary Table S2). The first group (I) had 38 DEGs that were upregulated in the G type and only 4 DEGs (ethylene-responsive transcription factor 2 (EVM0006642.1 and EVM0001843.1) and trihelix transcription factor GT-2 (EVM0022209.1 and EVM0024289.1)) that were more highly expressed in the G–O type group. The 5 DEGs in Group III were upregulated in sun leaves in both G- and NG-type germplasm resources. These genes encode ethylene-responsive transcription factor ERF016 (EVM0018364.1), transcription factor MYB1R1 (MSTRG.19007.2), dehydration-responsive element-binding protein 1C (MSTRG.5684.2), nuclear transcription factor Y subunit B-8 (MSTRG.8995.2) and WRKY transcription factor 50 (MSTRG.9491.24). Thirteen DEGs were upregulated in the NG–O type (IV) group: dehydration-responsive element-binding protein 1D (EVM0017184.1), dehydration-responsive element-binding protein 3 (EVM0020978.1), ERF4 (EVM0007694.1), Myb family transcription factor APL (EVM0001086.1), nuclear transcription factor Y subunit B-5 (MSTRG.251.1), WRKY 47 (MSTRG.445.4), scarecrow-like transcription factor PAT1 (EVM0020852.1), telomere repeat-binding factor 4 (EVM0009125.1), bHLH130 (MSTRG.18206.4), and bHLH35 (MSTRG.16167.1). Additionally, the expression of 31 DEGs in the second group (II) was upregulated in shade leaves in both G- and NG-type germplasm resources. The 23 DEGs in the fifth group (V) had higher expression in both sun and shade leaves of NG plants than G plants.

**Fig. 5:**
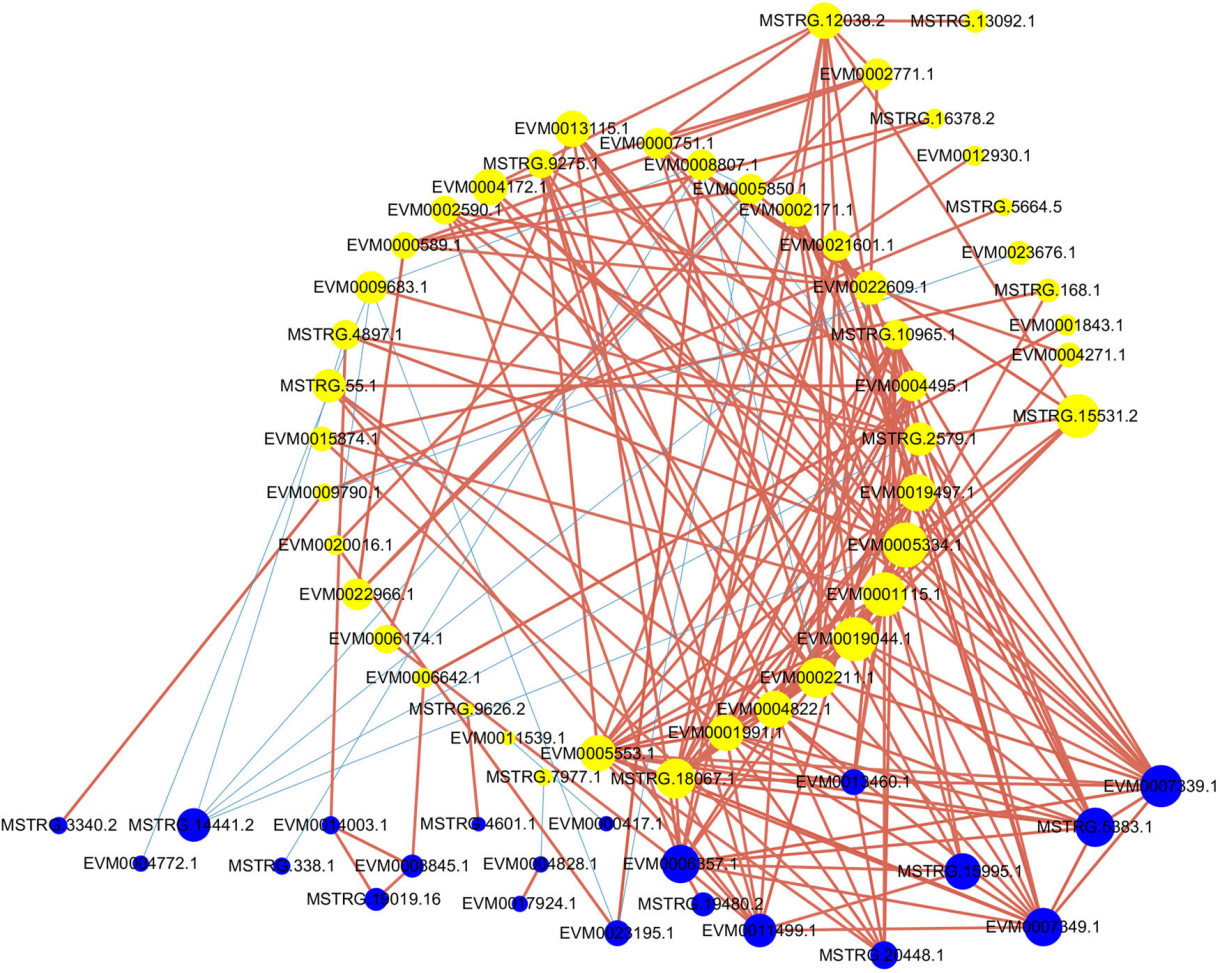
The coexpression network of DEGs involved in lipid metabolism and transcription factors. The yellow nodes represent TFs, and the blue nodes represent genes involved in lipid metabolism. The blue edges indicate a negative correlation, and the red edges indicate a positive correlation. The size of nodes is determined by the degree, which indicates connectivity. The width of edges is determined by the correlation

Next, a coexpression network of these DEGs was built based on their expression patterns. A total of 245 nodes and 29,646 coexpressed gene pairs were found. Then, the gene pairs with a *p*-value < 1e-5 (68 nodes and 204 edges) were considered to show consistent correlations and were used for coexpression network construction (Fig. 5 and Supplementary Table S3). As seen in Fig. 5, there were many interconnections among the TFs and DEGs involved in wax accumulation-associated pathways, which indicates that wax accumulation is a coordinated process. Among them, *WRKY40* (EVM0021601.1), *WRKY51* (MSTRG.2579.1), *WRKY70* (EVM0002590.1), *WRKY75* (MSTRG.18067.1 and EVM0019044.1), *bHLH35* (EVM0005334.1), *bZIP60* (EVM0004172.1), *MYB3* (EVM0013115.1), *MYB108* (EVM0019497.1), *MYB86* (EVM0005553.1), and *MYC2* (EVM0001115.1) had positive relations with nsLTP (nonspecific lipid-transfer protein-like protein, EVM0007339.1), which is involved in lipid transportation and cutin deposition^20^. Similar correlations were also found for *bHLH35* (EVM0005334.1), *MYB3* (EVM0013115.1), *ERF1B* (EVM0002171.1), and *KCS11* (3-ketoacyl-CoA synthase 11, MSTRG.15995.1), which are involved in very long-chain fatty acid and wax synthesis^21^. *PAS2A* (MSTRG.3340.2), which catalyzes the third reaction of the four-step cycle in the elongation of VLCFAs, was found to have an extremely significantly (*P* = 2.723E-6) positive relationship with *WRKY25* (EVM0009790.1)^22^. In addition, correlation values were found for TF–TF and TF–structural genes (Fig. 5). A given TF and its interconnected TFs should be considered when investigating the regulation of wax accumulation.

### qRT-PCR validation of differentially expressed genes

**Fig. 6:**
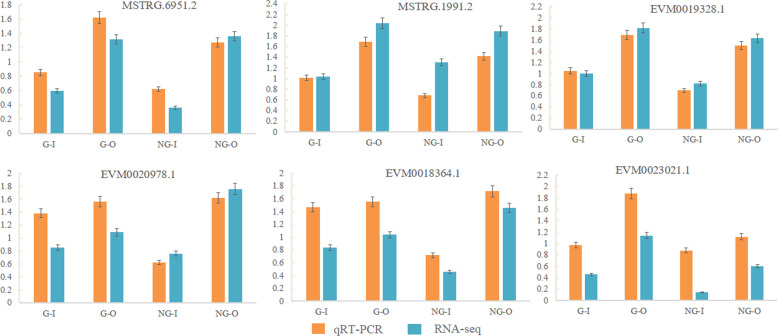
qRT-PCR expression profiles of 6 candidate genes. The data represent the means from three replicates with three biological repeats. Error bars indicate SEs

To further experimentally confirm the reliability of the transcriptome data, six DEGs were selected for qRT-PCR analysis. Four DEGs related to wax accumulation (*LACS8* (MSTRG.1991.2), lipid-transfer protein DIR1 (EVM0023021.1), ABC transporter G family member 14 *ABCG14* (EVM0019328.1), Ultraviolet-B receptor UVR8 (MSTRG.6951.2)), and 2 TFs (*MYB1R1* (EVM0018364.1) and *WRKY50* (EVM0020978.1)) were selected for validation. *LACS8*, *URV8* and *ABCG14* had higher expression in both G–O and NG–O types than in the G–I and NG–I types. *MYB1R1* and *WRKY50* were upregulated in the NG–O type compared with the NG–I type. While *DIR1* was upregulated in both G–O and NG–O types compared with G–I or NG–I types, its highest expression was in the G–O type (Fig. 6). The expression patterns of these DEGs were consistent with the RNA-seq results, further demonstrating the reliability of the sequencing results.

## Discussion

As one of the most important woody oil and biomass energy tree species in China, the study of yellowhorn drought resistance is becoming increasingly important. An increasing number of studies have revealed the drought tolerance of yellowhorn^14^; however, most of them focused on physiological indexes of drought resistance and relevant anatomical structures, such as leaf stomata and root characteristics. The molecular mechanisms of leaf epicuticular wax accumulation in yellowhorn still need to be investigated. In this study, we performed physiological and transcriptomic analyses of two types of yellowhorn germplasm resources, G type and NG type, which have contrasting epicuticular wax contents. Based on the comparative transcriptome data, the number of DEGs in different germplasm resources and under different conditions was identified, providing insights into the potential molecular mechanism underlying yellowhorn leaf epicuticular wax accumulation.

The combined analysis of leaf anatomy and cuticle properties showed that the epidermal wax content of yellowhorn was closely correlated with drought resistance, which is consistent with previous studies^23–25^. In addition, to further verify the correlation between wax thickness and WUE in yellowhorn, a linear relationship was established between the ^δ^13C value and wax thickness (*R*^2^ = 0.8457) in a larger group of 242 samples (unpublished data). The results indicated that the cuticular wax content is an important index for selecting drought-resistant germplasm resources.

In addition to its major role in stress response and adaptation, the cuticle is thought to play essential functions in plant interactions with the environment^26^. Additionally, the content and composition of cuticular wax vary greatly not only in different species but also under different environmental conditions, such as UV-B radiation, temperature and water conditions^27^. In this study, two types of yellowhorn germplasm resources differing in wax content were used in field trials. In addition, the sun leaves and shade leaves also had significant differences in both types because of their exposure to different temperature and UV-B radiation conditions, which is in line with previous studies. Surface waxes are effective reflectors of both UV and longer wavelength radiation^28^. Generally, sun leaves, from the outer part of the crown, have a higher wax accumulation to cope with higher temperatures and UV-B radiation. Robberecht, Caldwell and Billings demonstrated that plant epidermal UV-B attenuation was related to solar UV-B exposure along a latitudinal gradient, paying great attention to leaf properties^29^. Similarly, researchers also found that higher UV-B radiation could alter plant epicuticular wax formation and lipid metabolism in some crop plants^30^. Furthermore, determination of membrane lipid composition demonstrated that differences in the UV-B susceptibility of two germplasm resources may involve differences in lipid metabolism in cucumber^7,31^. Certainly, there is no doubt that analyzing wax constituents will provide new insights into the mechanisms regulating wax accumulation in future studies.

With the development of next-generation sequencing (NGS) technology, physiological and transcriptomic analyses have been used in several studies to reveal genetic expression^32–34^. Based on the significant differences in the wax contents and cuticular properties of sun and shade leaves, transcriptomic analyses were carried out. Several DEGs were found not only in different germplasm resources but also in different parts of the same tree. Overall, the number of DEGs in different germplasm resources was higher than that in different parts of the same tree due to their different genetic backgrounds. There was a strong genotype-by-environment interaction among the samples, which explains why the number of overlapping DEGs in the G–O vs. G–I and NG–O vs. NG–I comparisons was very small. Other studies have also found that genotype-by-environment interactions are common in other species and often caused by changes in the magnitude of genetic effects in response to the environment^35^. A genomics study in *A. thaliana* also revealed the major role of genotype-by-environment interactions in gene expression levels^36^. In addition, research on *Drosophila melanogaster* also found that a substantial fraction of the transcriptome exhibited genotype-by-environment interactions, suggesting an environmentally plastic genetic architecture of gene expression^37^. In this study, the G-type and NG-type leaves shared only a small percentage of DEGs under different light density conditions, which means that there are likely different regulatory mechanisms between G- and NG-type leaves under the different conditions that are affected by certain environment-specific promoters. Consequently, in breeding for drought resistance, not only should differences in genotype be considered but also environmental and genotype × environment interaction differences should be considered.

Studies on crops and *Arabidopsis* have revealed a large number of structural genes and synthesis pathways involved in cuticular wax biosynthesis and deposition on plant surfaces^8,38^. We also found that some UV stress response and lipid metabolism genes were expressed differently in different samples. The *Arabidopsis thaliana* protein UVR8, which triggers a signaling pathway for ultraviolet protection, is a photoreceptor for ultraviolet-B^18^. In our study, we found that *UVR8* (MSTRG.6951.2) was upregulated in both the G–O and NG–O types, especially in the G–O type. This result demonstrates that URV8 may be a signaling molecule that regulates the expression of genes involved in wax accumulation in yellowhorn leaves in response to ultraviolet-B radiation; its downstream substrate could be discovered by combining biochemical and molecular biological technologies in a follow-up study.

Gene expression pattern and enrichment analysis showed that, overall, the higher wax accumulation in the G-type than in the NG-type germplasm resources was due to more active key enzymes of lipid metabolism in the former. Alkane hydroxylase *MAH1* (EVM0012846.1), which catalyzes the production of secondary alcohols and their subsequent oxidation in ketones, was found to be upregulated in the NG–O type^39^. Long-chain acyl-CoA synthetase *LACS8* (long-chain acyl-CoA synthetase 8) was also upregulated in both the G–O and NG–O types, while *LACS7* was upregulated only in the G type (G–O and G–I types). However, LACS7 was found to have overlapping functions with LACS6, which is involved in oxidation in the peroxisome, while mutation tests in previous studies indicated that LACS8 and LACS9 proteins are not involved in wax synthesis^40,41^.

ABC transporters, such as ABCG11 and ABCG12, export wax molecules from the plasma membrane to the extracellular matrix^42^. Researchers have found that other ABC transporters, in addition to ABCG11 and ABCG12, are also needed to export wax compounds since 50% of the waxes on the plant surface remain in the absence of ABCG11 and ABCG12^43^. In addition, new studies show that ABCG9 and ABCG14 are plasma membrane-localized proteins that interact physically to form homo and/or heterodimers with ABCG11^44^. This conclusion supports the idea that there may be other transporters involved in wax accumulation. In this study, we found that ABC transporter G family member 14 (EVM0019328.1) was upregulated in both the G–O and NG–O types. This result provides clues about the potential transporters involved in wax transport. The DEG expression patterns showed that the DEGs identified in different types of germplasm resources were also different. There were 10 structural DEGs and 5 TFs highly expressed in G–O, while there were 4 structural DEGs and 13 TFs upregulated in NG–O. These results indicate that there may be different wax accumulation regulatory mechanisms in different types of germplasm resources that are activated in response to different light densities.

Recently, with wax biosynthetic pathways being elucidated, a great deal of studies have found that numerous transcription factors function as regulatory components of wax accumulation. Several members of the MYB transcription factor family, such as MYB30, MYB41, MYB94, and MYB96, were found to act as regulatory elements of cuticle synthesis in response to drought, ABA, cold and light stress^45–48^. These TFs also mediated different transcriptional regulation pathways. Guo et al. found that the overexpression of *MYB96* increases wax deposition by improving the expression of *LTP3*, which is a lipid transfer protein^48^. MYB30 is mainly involved in the regulation of gene expression related to the acyl-CoA enzyme complex^45^. In addition, some AP2/ERF transcription factors are also involved in activating cuticular wax biosynthesis in *Arabidopsis*^10,13,49^. In addition, the WW domain protein HD-ZIP (homeodomain-leucinezipper) and WRKY family transcription factor were also proposed as regulatory components of wax biosynthesis in *Arabidopsis* and rice^50^. These results demonstrate that there are several transcription factors that regulate cuticular wax accumulation, with multiple approaches to cope with complex stresses in the environment. However, the number of TFs that have been identified to regulate wax biosynthesis in a given plant is relatively small^8^. Moreover, the relationships among these TFs regulating wax accumulation have not been extensively explored. A great deal of unknown potential regulators need to be identified to understand the underlying mechanism of cuticular wax loading. The transcriptome sequencing data in this study provide a chance to discern regulatory networks and to screen new regulators involved in the wax accumulation pathway. In our study, some transcription factors were differentially expressed in different samples (Fig. 4). We also found that their expression patterns were significantly correlated (*p* < 1e-5) with those of structural genes in wax biosynthetic pathways based on the coexpression network (Fig. 5). These expression pattern correlations will provide a foundation and data for exploring the regulatory components and mechanisms of transcription.

## Conclusion

In conclusion, significant wax accumulation variation was found not only between germplasm resources but also in different parts of the same tree. Morphological and physiological comparisons indicated that the sun leaves from the outer part of the crown have a thicker epicuticular wax, which alters the permeability and improves the drought resistance of leaves, than shade leaves. Based on transcriptomic analysis, we identified several differentially expressed structural genes and transcription factors that are potentially involved in wax biosynthesis and transport. Most DEGs identified in this study showed different expression patterns in different types of yellowhorn germplasm resources in response to different light densities. These results propose a theoretical basis for screening and developing drought-resistant yellowhorn germplasm resources. Additionally, this work provides data for revealing the regulatory mechanism of wax loading and stress response in yellowhorn.

## Materials and methods

### Plant materials and growth conditions

Ten-year-old yellowhorn seedling trees at the Yellowhorn Germplasm Resources Garden at Liaoning, Zhangwu (122°520 E; 42°420 N), China, were selected as study materials; the trees were all subjected to the same management practices. We selected glossy (G)-type yellowhorn seedlings, whose mature leaves are dark green with glossy wax, and non-glossy (NG)-type yellowhorn plants, whose leaves are gray-green and not glossy, for their contrasting wax contents (Fig. 1a). Three plants were selected from both the G- and NG-type yellowhorn germplasm resources. Leaves from different positions of the same plants were also collected. Sun leaves (O) exposed to direct rays of the sun and sufficient light were selected from the outer part of the crown. The control shade leaves (I) on the same plants were collected from the lower or middle areas of the crown and were growing in dense shade. Light intensity was measured with a portable photosynthesis system (Li-Cor-6400; Li-Cor, Inc., Lincoln, NE, USA) in July 2018. In early July 2018, the key period of leaf epicuticular wax loading^51^, leaves of the two types of material were collected. Only fully expanded, undamaged leaves showing no signs of senescence were sampled from the southern aspect of branches. The leaves used for RNA sequencing were collected from each individual plant, frozen separately in liquid nitrogen, and stored at −80 °C.

### Physiological and anatomical analyses

Stable carbon (δ^13^C) isotope values of leaves were measured to examine long-term water use efficiency (WUE). Three replicates and 10 leaves per replicate were collected for each sample. The carbon isotope composition (δ^13^C) was estimated as follows:

δ^13^C = (Rsample/Rstandard-1) × 1000,

where Rsample and Rstandard are the ^13^C/^12^C ratios in a sample and the PBD (Pee Dee Belemnite) standard, respectively^52^.

To determine the wax contents of sun and shade leaves from each plant, the leaf epicuticular wax was extracted as described by Sen^53^. A total of 3–4 leaves were collected for each sample and scanned using a hand-held scanner (Epson, Beijing, China). Images were analyzed to estimate leaf area (LA) using IMAGEJ software^54^. Leaf epicuticular wax was extracted with 30 ml chloroform at room temperature (26 °C) with 1 min of gentle shaking. The leaves were rinsed using 5 ml chloroform, and the solution was decanted into a sample vial. After the chloroform evaporated, the mass of wax in the chloroform solution was weighed. The wax content was calculated as the extracted wax weight divided by the LA.

To evaluate the cuticle properties of sun leaves and shade leaves from different plants, the cuticular permeability was measured using the chlorophyll leaching method^55^. Four fronds were collected from each sample and kept in the dark for 16 h to ensure that the stomata were closed. The third expanding leaf from the top was sampled from each frond and immersed carefully in 50 ml of 80% ethanol at room temperature. At 20, 40, 60, 90, 120 min and 3 h, a 1 ml aliquot was removed for chlorophyll quantification and poured back to the same tube after quantification. Each sample had three replications. The chlorophyll concentration was quantified using a spectrophotometer at wavelengths of 663 and 647 nm and calculated using Lolles’ method^56^.

The water loss in detached leaves was also measured over a period of time. The fourth expanding leaf from the top was collected from each frond and immersed in 50 ml of deionized water for 3 h in the dark. The leaf surface moisture was gently wiped away, and leaf weights were measured every 20 min.

Anatomical characteristics of sample leaves were observed under a JEM-1230 electron microscope (JEOL, Tokyo, Japan). The sample leaves were gently washed with deionized water and fixed in FAA (70% ethyl alcohol, formaldehyde and glacial acetic acid V:V:V = 18: 1:1). Dehydration was performed using a series of ethanol and xylene with different concentration gradients, and leaves were then embedded in paraffin wax. Sections (10 µm thick) were dyed with safranine and fast green dyes and observed under a microscope. For each sample, five leaves were observed by light microscopy. Wax thickness was measured 30 times for each leaf. SPSS 22.0 statistical software was used for analysis. The quantitative data were tested by one-way ANOVA, and multiple comparisons between groups were performed with Tukey’s multiple comparisons test.

For scanning electron microscopy (SEM), the sun leaves and shade leaves from different plants were detached and then stored in 5% glutaraldehyde fixative at 4 °C. For each sample, five leaves were observed using SEM. The samples were transferred to an ethanol series with different concentration gradients in a supercritical dryer (Tousimis, Autosamdri-815, America) for at least 4 h for dehydration. After the samples were coated with gold by an ion sputter coater (Hitachi MC 10003, Japan), the adaxial surfaces of leaves were subsequently observed using a scanning electron microscope (Hitachi SU8020, Japan) with an accelerating voltage of 5 kV.

### RNA extraction and sequencing

Three ten-year-old yellowhorn seedling trees were selected from both G- and NG-type germplasm resources. Twelve libraries were created from three biological replicates each for sun leaves (O) and shade leaves (I) of the G- and NG-type germplasm resources. Twenty fresh and mature leaves from each biological replication were collected and frozen in liquid nitrogen immediately. RNA from sun leaves and shade leaves from different germplasm resources was extracted using an RNAprep Pure Plant Kit (Polysaccharides- and Polyphenolics-rich) (Tiangen Biotech, Beijing, China, No. DP441) according to the manufacturer’s instructions. The concentration of each RNA sample was checked using a NanoDrop (Thermo Fisher Scientific Inc., USA) and QUBIT ® Fluorometer (Life Technologies). RNA integrity was checked using a Bioanalyzer 2100 (Agilent Technologies). cDNA library construction was carried out following the manufacturer’s instructions (Illumina, San Diego, CA, USA) and sequenced by Annoroad Co., Ltd. (Beijing, China) using an Illumina HiSeq platform (Illumina, San Diego, CA, USA); 2 × 150 bp paired-end reads were generated.

### DEG and enrichment analyses

After removing Illumina adapters and reads with unknown nucleotide contents >5% and trimming low-quality reads (more than 50% of bases in the total reads had a quality score lower than 19), the remaining high-quality reads (clean reads) with an average length of 150 bp were used in this study. The yellowhorn genomes in our previous study were used as references^1^. The genome index was built, and the clean reads were mapped to the reference genome using hisat2^15^. The expression levels of the genes in each sample were assessed using fragments per kilobase per million mapped reads (FPKM) values. The number of reads for each gene in the samples was counted by StringTie^15^. We detected the DEGs between 4 samples using Ballgown packages. Based on *p* < 0.05 and differential expression |log2_FC | ≥ 1, DEGs between two samples were identified.

GO and KEGG enrichment analyses of DEGs was performed by the Omicshare online program (https://www.omicshare.com/tools/), and GO terms with corrected *p* < 0.05 were considered to be significantly enriched by DEGs. Pearson’s correlation coefficients were used to measure coexpression relationships by the Omicshare online program (https://www.omicshare.com/tools/), and the Cytoscape tool was used to construct a coexpression network^57^.

### Reverse transcription and qRT-PCR

The relative expression levels of individual genes were measured with gene-specific primers by real-time quantitative PCR (qRT-PCR) analysis to verify the correctness of the expression of these candidate genes. RNA (1 µg) was treated with DNaseI and reverse-transcribed with oligo (dT) using a PrimeScriptTMRT Reagent Kit (Takara, Japan). The gene-specific primers were designed by Primer6. The qRT-PCR experiment was performed using SYBRGreen Fast qPCR Master Mix (Sangon Biotech, China) on an ABI StepOne Plus Real-Time System (ABI, USA) according to the manufacturer’s protocols. Each PCR (total volume of 20 µL) contained 10 µL of SYBR Green mix (TOYOBO, Osaka, Japan), 1 µL of each primer (50 pmol), 2 µL of template cDNA and 6 µL of ddH2O. The amplification program included 95 °C for 3 min, followed by 95 °C for 10 s, 60 °C for 30 s, and 72 °C for 3 s (45 cycles). There were three biological replicates for each gene. The *Arabidopsis* homolog of β-actin was used as an internal reference gene to normalize the qRT-PCR expression data. The relative mRNA abundance in samples of each gene was calculated using the 2^−ΔΔ^Ct method^58^. The primers used in this study are listed in Supplementary Table S4.

## Supplementary information

Response Additional File3

Supplementary FigS1

Supplementary FigS2

Supplementary FigS3

Supplementary FigS4

Supplementary TableS1

Supplementary TableS2

Supplementary TableS3

Supplementary TableS4

Response Additional File1

Response Additional File2

## Data Availability

Raw Illumina sequence data were deposited in the National Center for Biotechnology Information (NCBI) and can be accessed in the sequence read archive (SRA) database (https://www.ncbi.nlm.nih.gov/sra). The accession number is PRJNA676181 and includes 12 accession items (SRR13038199-SRR13038210). All data generated or analyzed during this study are included in this published article and its supplementary information files.
