# Supplementary figures and images for: Transcriptome and physiological analyses provide insights into the leaf epicuticular wax accumulation mechanism in yellowhorn
Source: Hortic Res. 2021 Jun 1;8:134. doi: 10.1038/s41438-021-00564-5 (PMC8167135; doi:10.1038/s41438-021-00564-5)

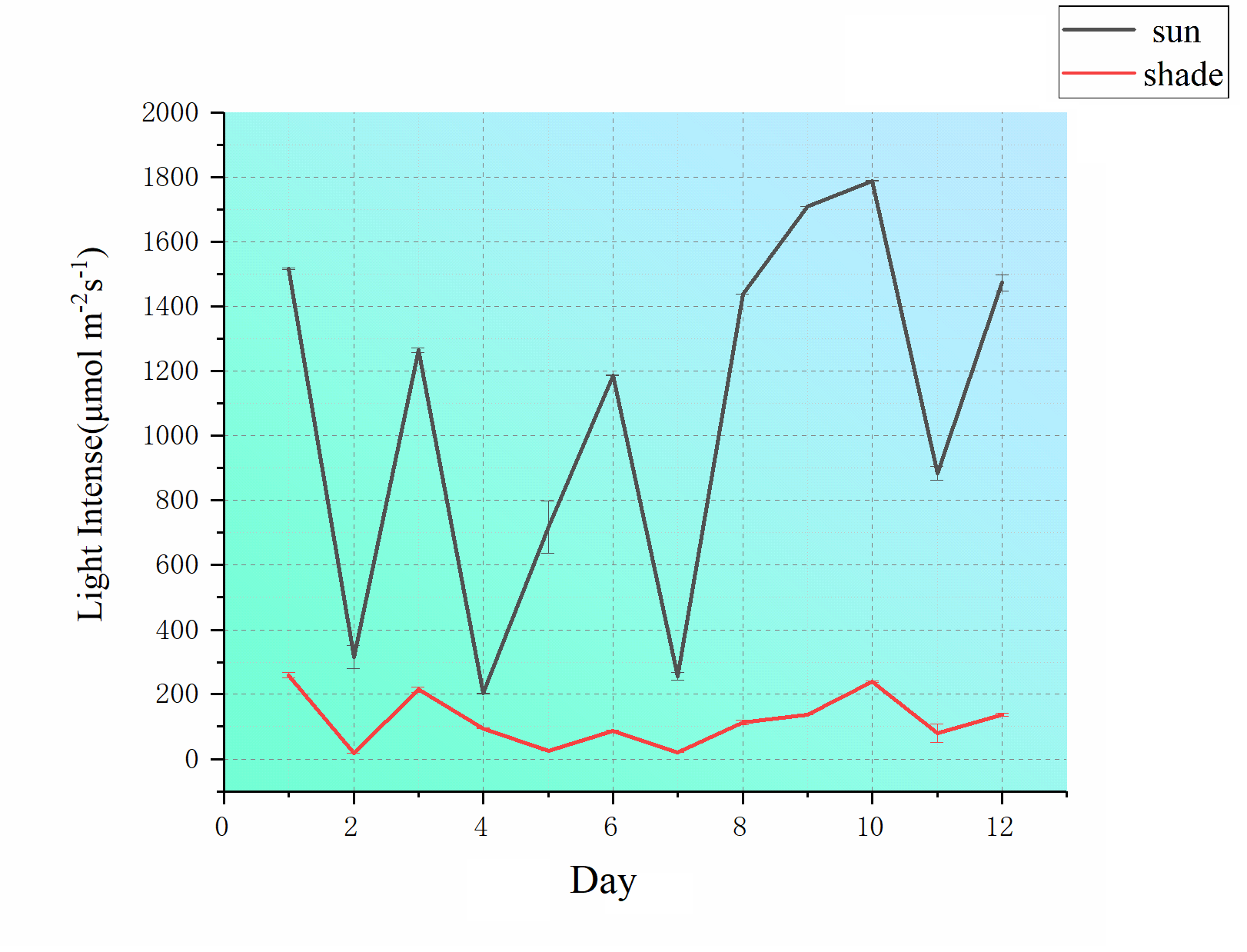

Supplement: Supplementary file 2 — Supplementary FigS1 [file 41438_2021_564_MOESM2_ESM.tif]

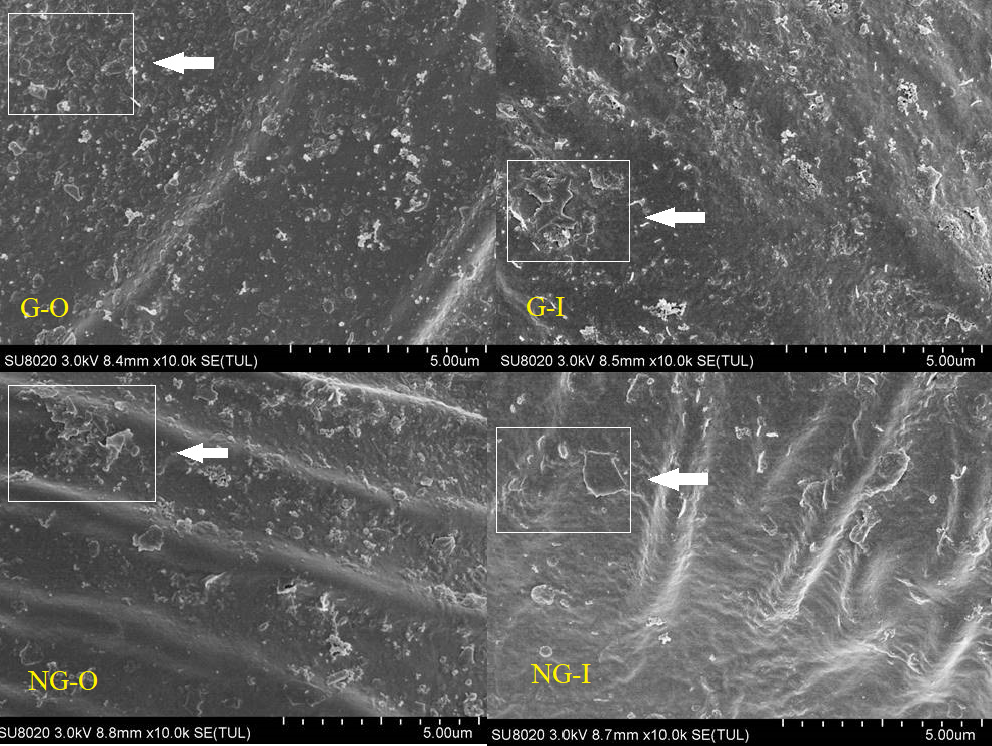

Supplement: Supplementary file 3 — Supplementary FigS2 [file 41438_2021_564_MOESM3_ESM.tif]

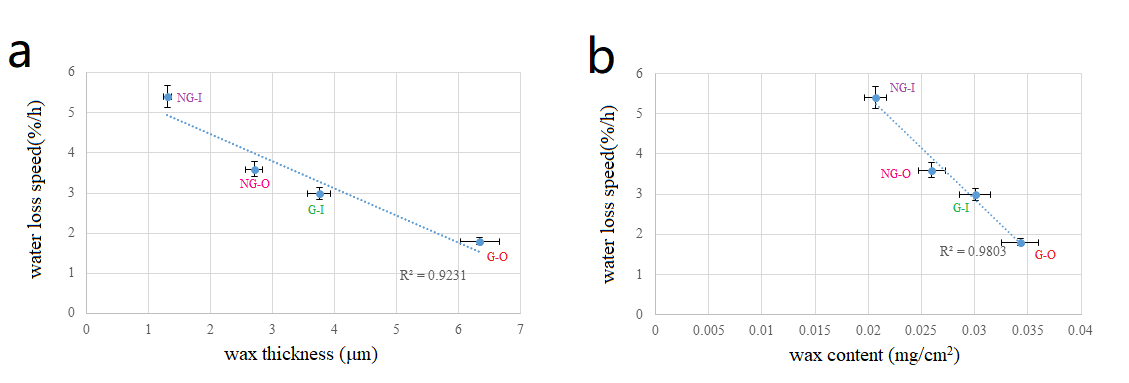

Supplement: Supplementary file 4 — Supplementary FigS3 [file 41438_2021_564_MOESM4_ESM.tif]

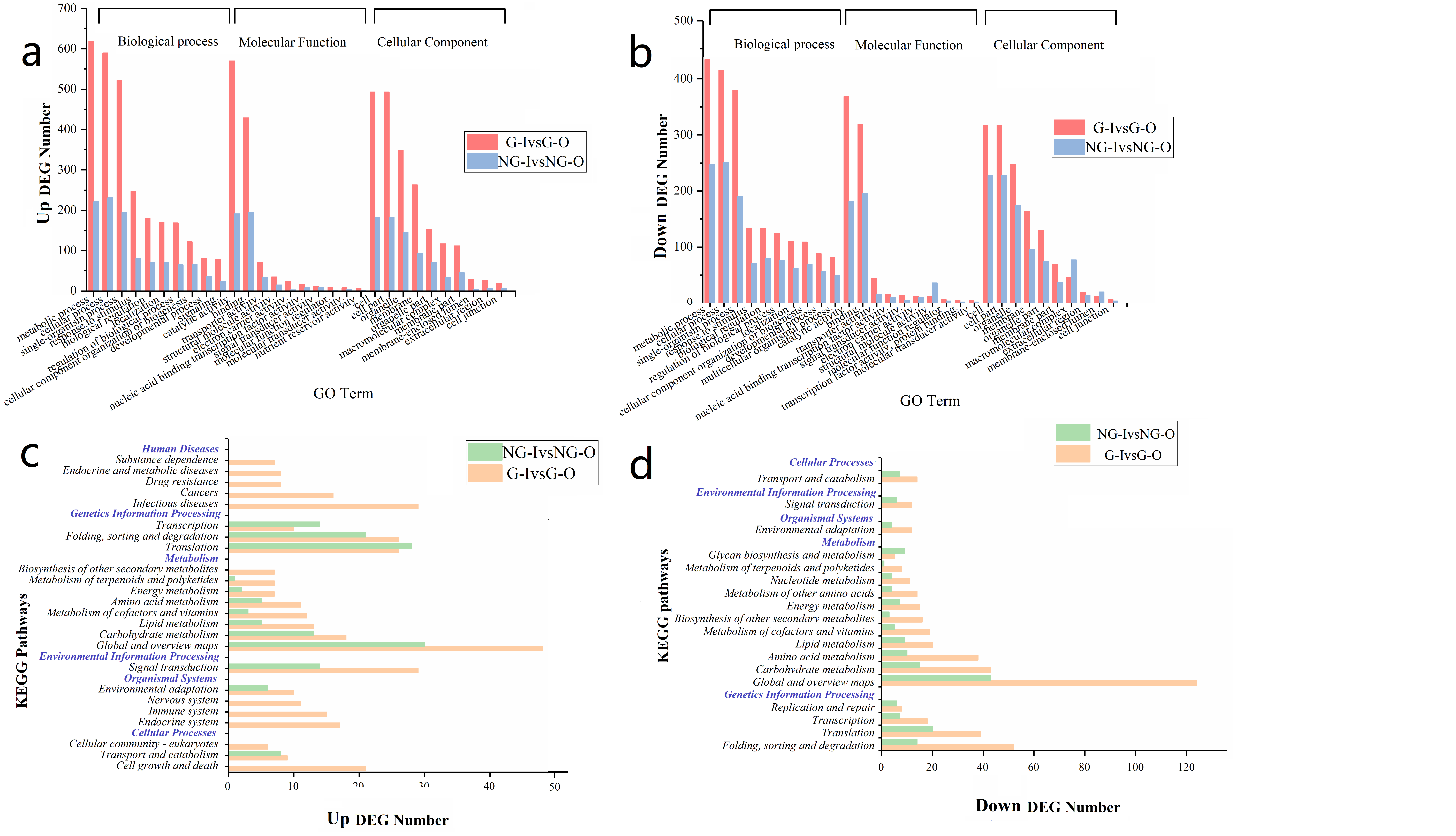

Supplement: Supplementary file 5 — Supplementary FigS4 [file 41438_2021_564_MOESM5_ESM.tif]

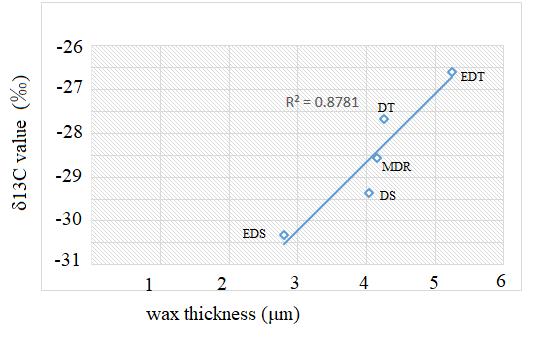

Supplement: Supplementary file 11 — Response Additional File2 [file 41438_2021_564_MOESM11_ESM.tif]
